# Keeping in shape

**DOI:** 10.7554/eLife.20468

**Published:** 2016-09-13

**Authors:** Craig Blackstone, William A Prinz

**Affiliations:** 1 National Institute of Neurological Disorders and Stroke, National Institutes of Health, Bethesda, United StatesBlackstC@ninds.nih.gov; 2 National Institute of Diabetes and Digestive and Kidney Diseases, National Institutes of Health, Bethesda, United States

**Keywords:** endoplasmic reticulum, organelle morphology, membrane structure, Human, *Xenopus*

## Abstract

Three proteins work together to control the shape of the endoplasmic reticulum in animal cells.

**Related research article** Wang S, Tukachinsky H, Romano FB, Rapoport TA. 2016. Cooperation of the ER-shaping proteins atlastin, lunapark, and reticulons to generate a tubular membrane network. *eLife* **5**:e18605. doi: 10.7554/eLife.18605

The endoplasmic reticulum is the largest single structure in eukaryotic cells. It consists of a range of interconnected shapes, including sheets and tubules, and comprises a lumen enclosed by a membrane that is continuous with the membrane that surrounds the nucleus of the cell (Figure 1). The structure and dynamic nature of the endoplasmic reticulum allow it to be involved in many processes in cells: these processes include protein production and degradation, cell signaling, and the synthesis and distribution of lipids and fat molecules. Form follows function, and understanding how the distinct shapes of the endoplasmic reticulum are regulated and maintained is currently an area of intense interest in cell biology (Goyal and Blackstone, 2013; Westrate et al., 2015).

**Figure 1.**
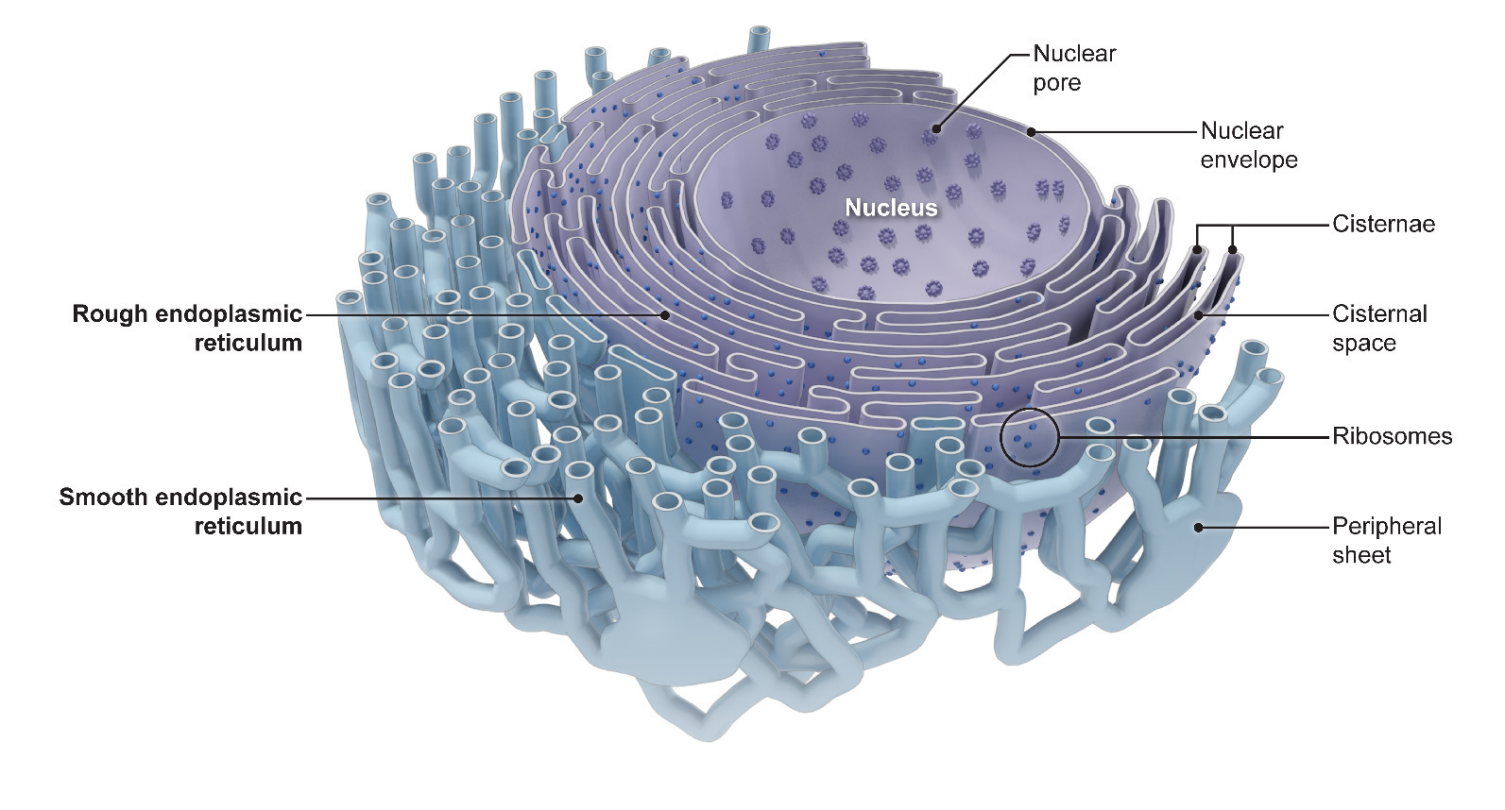
The endoplasmic reticulum consists of various interconnected shapes. At the center of the cell, the nuclear envelope contains pores that control what molecules enter and exit the nucleus. The nuclear envelope is also connected to the stacked sheets (cisternae) of the rough endoplasmic reticulum, which is specialized for protein production. From the rough endoplasmic reticulum, the tubules of the smooth endoplasmic reticulum (blue) form a network that extends across the cell and is interspersed with sheet-like structures (peripheral sheets). From Goyal and Blackstone (2013).

Image credit: Goyle and Blackstone (public domain).

Over the past decade, several proteins that shape the endoplasmic reticulum have been identified. In many cases, these proteins are evolutionarily conserved across eukaryotes, from yeast to mammalian cells. Membrane proteins of the reticulon and REEP families can generate curves in membranes and act to maintain the tubules (Voeltz et al., 2006). Atlastin proteins mediate the tethering and fusion of tubules to one other to form three-way junctions (Hu et al., 2009; Orso et al., 2009), which appear to be stabilized by a membrane protein called lunapark (Shemesh et al., 2014; Chen et al., 2015). Several other proteins help the endoplasmic reticulum to maintain contact with the cell membrane, other cell compartments and the cytoskeleton. Increasingly, studies have revealed dynamic changes in the shape of the endoplasmic reticulum in processes such as cell division and during electrical activity in neurons (Goyal and Blackstone, 2013; Phillips and Voeltz, 2016).

Proteins involved in shaping the endoplasmic reticulum have mostly been studied individually, even though they are known to interact with one another. Now, in *eLife*, Tom Rapoport and co-workers at Harvard Medical School – including Songyu Wang, Hanna Tukachinsky and Fabian Romano – report on how three key proteins work together to shape and maintain the endoplasmic reticulum (Wang et al., 2016).

Wang et al. performed CRISPR/Cas9 gene knock outs and stable gene transfections in mammalian cells and also investigated egg extracts from the frog *Xenopus*, which can form an endoplasmic reticulum network in vitro that is strikingly similar to that seen in intact cells. They found that in addition to being required for the formation of three-way junctions, atlastins are also necessary to maintain such junctions. Wang et al. further report on the interplay among the proteins that are involved in shaping the endoplasmic reticulum. For instance, lunapark is not required for three-way junctions to form, but its depletion appears to cause a loss of tubule junctions and an increase in the number of sheet-like structures.

Another remarkable finding is that the endoplasmic reticulum network fragments if atlastin is inhibited (see also Orso et al., 2009), or if the reticulon proteins are overexpressed. This indicates that the network can spontaneously disassemble in some circumstances and may explain why no proteins specifically involved in the splitting of tubules have ever been identified. Although the endoplasmic reticulum is generally thought to be continuous, previous studies have shown that it can split up in certain situations, for example during the fertilization of starfish eggs or during excessive electrical activity in neurons (Goyal and Blackstone, 2013). A future challenge will be to find out how and why cells might fragment their endoplasmic reticulum.

Finally, Wang et al. propose a compelling mechanism for how lunapark is regulated by phosphorylation during cell division. Modifying lunapark to mimic phosphorylated lunapark caused it to disappear from three-way junctions. This result, coupled with a recent study showing that lunapark is a component of a ubiquitin ligase complex at three-way junctions (Zhao et al., 2016), will probably lead to additional studies into how structural modifications regulate these proteins to control the shape of the endoplasmic reticulum.

We have likely just scratched the surface of how the endoplasmic reticulum is shaped, and additional proteins and regulatory mechanisms will surely be uncovered. Investigating the dynamic interactions of the endoplasmic reticulum with other cell compartments and the plasma membrane seems a particularly exciting area. Furthermore, numerous endoplasmic reticulum shaping proteins are mutated in inherited neurological disorders, particularly the hereditary spastic paraplegias (Blackstone, 2012). Future studies will benefit from emerging new super-resolution microscopy tools, improving our understanding of how the endoplasmic reticulum is dynamically shaped in health and disease.
